# Discriminating Microbial Community Structure Between Peri-Implantitis and Periodontitis With Integrated Metagenomic, Metatranscriptomic, and Network Analysis

**DOI:** 10.3389/fcimb.2020.596490

**Published:** 2020-12-11

**Authors:** Keiji Komatsu, Takahiko Shiba, Yasuo Takeuchi, Takayasu Watanabe, Tatsuro Koyanagi, Takashi Nemoto, Masahiro Shimogishi, Masaki Shibasaki, Sayaka Katagiri, Shohei Kasugai, Takanori Iwata

**Affiliations:** ^1^ Department of Periodontology, Graduate School of Medical and Dental Sciences, Tokyo Medical and Dental University, Tokyo, Japan; ^2^ Department of Chemistry, Nihon University School of Dentistry, Tokyo, Japan; ^3^ Oral Implantology and Regenerative Dental Medicine, Tokyo Medical and Dental University, Tokyo, Japan

**Keywords:** peri-implantitis, periodontitis, metagenome, metatranscriptome, oral microbiome, dysbiosis

## Abstract

Peri-implantitis and periodontitis are both polymicrobial diseases induced by subgingival plaque accumulation, with some differing clinical features. Studies on the microbial and gene transcription activity of peri-implantitis microbiota are limited. This study aimed to verify the hypothesis that disease-specific microbial and gene transcription activity lead to disease-specific clinical features, using an integrated metagenomic, metatranscriptomic, and network analysis. Metagenomic data in peri-implantitis and periodontitis were obtained from the same 21 subjects and metatranscriptomic data from 12 subjects were obtained from a database. The microbial co-occurrence network based on metagenomic analysis had more diverse species taxa and correlations than the network based on the metatranscriptomic analysis. *Solobacterium moorei* and *Prevotella denticola* had high activity and were core species taxa specific to peri-implantitis in the co-occurrence network. Moreover, the activity of plasmin receptor/glyceraldehyde-3-phosphate dehydrogenase genes was higher in peri-implantitis. These activity differences may increase complexity in the peri-implantitis microbiome and distinguish clinical symptoms of the two diseases. These findings should help in exploring a novel biomarker that assist in the diagnosis and preventive treatment design of peri-implantitis.

## Introduction

Dental implants are the most popular treatment option for tooth loss from multiple causes, including periodontitis, to improve and support patient quality of life (Sonoyama et al., 2002). Peri-implant complications are mainly caused by peri-implantitis and can lead to dental implant loss (Roos-Jansaker et al., 2006). Periodontitis and peri-implantitis are defined as polymicrobial diseases caused by multiple bacterial species (Leonhardt et al., 1999; Koyanagi et al., 2010; Abusleme et al., 2013; Koyanagi et al., 2013; Maruyama et al., 2014; Zheng et al., 2015; Yu et al., 2019). There is strong evidence suggesting that a history of periodontitis is a risk factor for peri-implantitis (Fu and Wang, 2020). In addition, peri-implantitis shows clinical symptoms similar to those of periodontitis (Lindhe et al., 1992). However, peri-implantitis progresses more rapidly than periodontitis, and lesions of peri-implantitis extend into the bone marrow (Lindhe et al., 1992). Additionally, clinical treatments for peri-implantitis, including conventional periodontal disease treatment, are often ineffective (de Waal et al., 2016; Toma et al., 2019). The microbiome of peri-implantitis is often compared to that of periodontitis; most previous studies that focused on the taxonomic composition of the microbiome primarily used 16S ribosomal DNA (16S rDNA) sequencing (Maruyama et al., 2014; Schaumann et al., 2014; Yu et al., 2019). We previously reported that although the taxonomic and functional profiles were similar, co-occurrence network structure and core species taxa based on co-occurrence relationships were dissimilar between peri-implantitis and periodontitis in metatranscriptomic analysis which can reveal microbial ecological dynamics and identify core bacteria *in situ* (Shiba et al., 2016). However, when evaluating the activity of each gene, it is necessary to consider the quantities of each gene and transcript. Recent studies reported that the relative activity levels of different species in a microbial community can be revealed by comparing metagenomic and metatranscriptomic analyses (Yu and Zhang, 2012). Although this comparative analysis has been applied in some periodontitis studies (Duran-Pinedo et al., 2014; Belstrom et al., 2017), metagenomic analysis has not been conducted on the peri-implantitis microbial community before.

Therefore, we first performed a metagenomic analysis of peri-implantitis to explore the microbial and gene potential using DNA and combined it with metatranscriptomic data to determine the difference in microbial and gene activity between the two diseases.

## Materials and Methods

### Ethical Statement

This study was performed in accordance with the Ethical Guidelines for Clinical Studies (2008 Notification Number 415 of the Ministry of Health, Labor, and Welfare) and was approved by the Ethics Committee of Tokyo Medical and Dental University (D2015-535). All subjects provided written, informed consent prior to participating. The study was conducted according to the principles of the most recent Declaration of Helsinki.

### Subjects and Clinical Examination

Twenty-one patients with both peri-implantitis (dental implant functioning for >1 year) and periodontitis were recruited from the Tokyo Medical and Dental University Hospital Faculty of Dentistry from 2012 to 2018. Subject recruitment criteria were systemically healthy, non-smokers (including former smokers), and had not received antibiotics or anti-inflammatory drugs within the last 3 months. The following clinical parameters were recorded: probing depth (PD), radiographic bone loss (RBL), bleeding on probing (BOP), suppuration (SUP), and presence of keratinized mucosa (KM) measured as ≥2 mm/0–2 mm. The type of implant system and implant function time was also recorded at the peri-implantitis sites. The sites of peri-implantitis were defined as those having RBL ≥3 mm and/or probing depths ≥6 mm, with BOP and/or SUP, according to the latest peri-implantitis diagnostic criteria (Renvert et al., 2018). Originally, the sites of periodontitis were defined as those having a PD ≥4 mm, with the presence of RBL, and BOP and/or SUP (Koyanagi et al., 2010; Schaumann et al., 2014; Zhuang et al., 2016). All periodontal teeth were re-diagnosed based on RBL according to the current periodontitis diagnostic criteria (Tonetti et al., 2018) and diagnosed as Stages II–IV. To evaluate PD and BOP and/or SUP, metal periodontal probes (Hu-Friedy, Chicago, IL, USA) were used at the periodontitis sites. However, plastic periodontal probes (Hu-Friedy, Chicago, IL, USA) were used at the peri-implantitis sites to avoid damaging the implant surface. The moving implant as a result of complete loss of osseointegration and the implant that had not functioned much for at least 1 year were excluded from the study. RBL was evaluated by KK. The details of radiographic examination are described in the **Supplementary Methods S1**. Other periodontal examinations were performed by trained periodontists, specifically TS and TK in the Department of Periodontology at Tokyo Medical and Dental University.

### Sample Collection and DNA Extraction

Periodontal teeth and peri-implantitis-affected dental implants were isolated using sterile cotton rolls. Supra-gingival plaque was removed, and five sterile paper points were inserted into the deepest part of the periodontal pocket or implant sulcus for 30 s to collect subgingival plaque samples. Subgingival plaque samples were placed in sterile 1.5 ml tubes and stored at −80°C. DNA was extracted from samples using a bacterial DNA extraction kit (Mora-extract, AMR Inc., Tokyo, Japan) according to the manufacturer’s instructions. Extracted DNA quantity and quality were assessed using a Quantus Fluorometer (Promega, Madison, WI, USA) and Agilent 2100 Bioanalyzer (Agilent Technologies, Santa Clara, CA, USA) as previously described (Maruyama et al., 2014). The purity of DNA was assessed using Nano Drop Lite (Thermo Fisher Scientific, Waltham, MA, USA), and the samples that showed A260/280 ratio of around 1.8 were included for this study.

### Sample Preparation and Data Analysis for 16S rDNA Sequencing

Using the MiSeq platform (Illumina, San Diego, CA, USA), 16S rDNA sequencing was performed running 2 × 300 bp paired‐end reads targeting the hypervariable region of V3-V4. Library preparation was performed according to the Illumina 16S sample preparation guide (16S Sample Preparation Guide, Illumina). The 16S rDNA sequencing reads were processed according to the recommended parameters of the Illinois Mayo Taxon Organization from the RNA Dataset Operations (IM-TORNADO) (Jeraldo et al., 2014) pipeline for 300 bp reads. In the pipeline, each operational taxonomic unit (OTU) was assigned at the species level using the Human Oral Microbial database (Chen et al., 2010) at 97% sequence identity. The read counts were normalized by conversion to reads per million (RPM) (Shiba et al., 2016; Chen et al., 2019). The species name was slightly adjusted in order to be consistent with other databases. The details of sample preparation and parameters for data analysis are described in the **Supplementary Methods S2**. 16S rDNA sequence data were deposited in the DNA Data Bank of Japan (DDBJ; http://www.ddbj.nig.ac.jp/) under accession number DRA010104.

### Sample Preparation and Data Analysis for Metagenomic Sequencing

Metagenome data were obtained using a MiSeq V2 reagent kit (Illumina, San Diego, CA, USA) running 2 × 250 bp paired-end reads. Metagenomic library preparation was performed using a Nextera XT DNA library preparation kit (Illumina Inc., San Diego, CA, USA) according to the manufacturer’s protocol. Metagenomic sequencing data preprocessing was conducted according to a previous report (Shiba et al., 2016). Briefly, the paired and unpaired reads were assigned based on the Metagenomics Rapid Annotation using the Subsystem Technology (MG-RAST) pipeline (Meyer et al., 2008) to reveal the pathway profile with the Kyoto Encyclopedia of Genes and Genomes (KEGG) database (Kanehisa and Goto, 2000). The clusters which were estimated to be coding domain sequence CDS were assigned based on the Virulence Factors Database (VFDB; as of December 23, 2016) and the Microbial Virulence Database (MvirDB; as of December 20, 2016) to reveal the microbial virulence factors. We also examined the genetic profile and mRNA-derived bacterial species using the CDS clusters assigned based on the National Center for Biotechnology Information Non-Redundant Protein Database (NCBI nr; as of January 10, 2017). The read counts were normalized by conversion to reads per kilobase per million (RPKM) (Shiba et al., 2016; Yan et al., 2016; Chen et al., 2019; Cornejo-Castillo and Zehr, 2020; Javdan et al., 2020; Wei et al., 2020). The details of sample preparation and parameters for data analysis are described in the **Supplementary Methods S3**. Metagenomic sequencing data were deposited in the DNA Data Bank of Japan (DDBJ; http://www.ddbj.nig.ac.jp/) under accession number DRA006832.

### Co-Occurrence Network Construction Using Metagenome and Metatranscriptome Data

In order to exclude infrequent species from the co-occurrence network construction, only species present in at least 50% of samples detected by DNA (16S rDNA and metagenome) analysis and RNA (16S rRNA reads and metatranscriptome) analysis in the same subject were adopted for the following analysis. This criterion for determining species taxa was more stringent than that used by Shiba et al. (2016). Metatranscriptomic data were obtained from the DDBJ (DRA003492) (Shiba et al., 2016). The details of the data analysis of metatranscriptome are shown in **Supplementary Method S4**. Then we calculated the RNA (metatranscriptome)/DNA (metagenome) ratio to identify highly active species in each disease. Co-occurrence coefficients were calculated based on CDS and mRNA read abundances using the sparse correlations for the compositional data algorithm (SparCC) program (Friedman and Alm, 2012). Ten iterations were used to estimate the median correlation of each pairwise comparison. The statistical significance of each correlation was calculated by bootstrapping with 500 iterations (Milici et al., 2016). Network structures were constructed using two species taxa with a positive correlation in CDS or mRNA read abundance with SparCC values ≥0.4. Our criterion for significance testing was more stringent than the previously used value of ≥0.3 (Shiba et al., 2016). Co-occurrence patterns were drawn using a network structure in which each taxon and co-occurrence was indicated by a node and edge, respectively, for all taxon pairs with a positive correlation. Networks were visualized using Cytoscape software v.2.860 (Smoot et al., 2011).

### Pathway and Virulence Factor Profile Comparison Between the Metagenome and Metatranscriptome

Pathway and virulence factor profiles were compared based on KEGG, VFDB, and MvirDB using linear discriminant analysis effect size (LEfSe) analysis (Segata et al., 2011) to determine which genes were enriched in either the metagenome or metatranscriptome.

### Statistical Analysis

The numbers of raw and processed read counts and clinical parameters associated with peri-implantitis and periodontitis were compared using a two-tailed paired *t*-test. Wilcoxon’s signed-rank test was performed to test for significant differences in each taxon of genes between the two groups. The correlations between the clinical indices and the taxonomic read abundances were evaluated by Spearman’s rank correlation coefficient test. An analysis of similarity (ANOSIM) was used for testing the significance of dissimilarity between the two groups by applying the read abundance. A *P*-value was provided by performing a permutation test, which was used to evaluate the statistical significance of the calculated R-values in the ANOSIM. LEfSe analysis was used for comparison of both the metagenome and metatranscriptome. The threshold on the logarithmic linear discriminant analysis score for discriminative features was set to 3.5, and Kruskal–Wallis and Wilcoxon tests significance were used. This criterion was more stringent than the default parameter (linear discriminant analysis score 2.0). In all the statistical tests performed in this study, *P*-values <0.05 were considered to indicate statistical significance, and Benjamin and Hochberg’s false discovery rate was applied for multiple testing and *q <*0.1 was considered statistically significant.

## Results

### Subject Clinical Characteristics and Summary of Sequence Reads

Twenty-one subjects (12 males, 9 females; 67.2 ± 7.8 years, mean ± standard deviation) who were affected with both peri-implantitis and periodontitis in the same oral cavity were recruited in this study. Five of 21 subjects were former smokers. Mean PD of sampled sites in peri-implantitis and periodontitis were 7.7 ± 2.4 and 6.5 ± 2.3 mm, respectively. There were no significant differences in the following clinical parameters for the two disease sites: PD, BOP, SUP, RBL, and position (**Table 1**). Details of clinical parameters (including presence of KM, prosthesis types, and connection types) and sequence reads of each subject are available in **Supplementary Results S1** and **Supplementary Tables S1**, **2**. The details of clinical parameters associated with the metatranscriptomic analysis of 12 subjects who were enrolled in a previous report (Shiba et al., 2016) are available in **Supplementary Table S3**.

**Table 1:** Clinical characteristics of the study subjects.

		Peri-implantitis	Periodontitis	P value (two-tailed paired t test)
Age		67.2 ± 7.8^a^		–
Gender		12 males, 9 females		–
Sample sites	Maxilla	11	10	0.33
	Mandible	10	11	0.33
	Anterior	3	6	0.08
	Posterior	18	15	0.08
PD (mm)		7.7 ± 2.4^a^	6.5 ± 2.3^a^	0.11
Number of sites with BOP		21	21	–
Number of sites with SUP		3	0	0.08
RBL (%)		33.2 ± 13.6^a^	32.9 ± 13.3^a^	0.94
Time in function (year)		7.5 ± 4.9^a^	–	–

a Values represent the mean ± standard deviation. PD, probing depth; BOP, bleeding on probing; SUP, suppuration; RBL, radiographic bone loss.

### Taxonomic Profile and Diversity Based on 16S rDNA Sequencing

Rarefaction curve results demonstrated that all samples yielded a sufficient number of reads for 16S rDNA analyses (**Supplementary Figure S1A**). A total of 357.9 ± 110.2 and 319.1 ± 86.6 OTUs were identified in peri-implantitis and periodontitis samples, respectively. The Shannon index of peri-implantitis was significantly higher than that of periodontitis, whereas the number of OTUs did not significantly differ between the two diseases (**Supplementary Figures 1B, C**). The principal coordinates analysis (PCoA) based on 1-Spearman’s coefficient showed a similar beta diversity between the two diseases (**Supplementary Figure 1D**). The ANOSIM evaluation revealed that the microbial compositions of both diseases were similar (*R* = 0.021 and *P* = 0.199 at the species level). There was no significant difference in 16S rDNA read abundance between the two diseases using the Wilcoxon signed-rank test (**Supplementary Results S2**, **Supplementary Table S4**, and **Supplementary Figure S2**).

### Taxonomic Profiles Based on Metagenomic Analysis

We identified CDS-derived taxa by our previously reported method using the NCBI nr database (Funahashi et al., 2019). PCoA plots and ANOSIM indicated that the two diseases had a similar CDS-derived taxa composition (*R* = −0.006 and *P* = 0.523) (**Supplementary Figure S3A**). Wilcoxon tests revealed no differences in the CDS abundance of taxa between the two diseases (**Supplementary Results S3**, **Supplementary Table S5**, and **Supplementary Figure S3B**).

### Functional Potential Profiles Based on Metagenomic Analysis

Using the NCBI nr database, PCoA plots had similar CDS profiles for the two diseases (**Supplementary Figure S3C**). An ANOSIM revealed the similarity between the two groups (*R* = −0.010 and *P* = 0.659), and Wilcoxon tests showed no differences in the read abundance of CDS clusters or CDS abundances for any of the genes between the two diseases (**Supplementary Results S4** and **Supplementary Tables S6**, **7**).

### Pathway and Virulence Factor Profiles Based on Metagenomic Analysis

The MG-RAST (Meyer et al., 2008) pipeline analysis revealed the characteristics of putative CDS reads based on KEGG pathways. Wilcoxon tests revealed no differences in KEGG Level 4 abundance between the two diseases, which shared most metabolic pathways (**Supplementary Figure S4A** and **Supplementary Table S8**). PCoA and ANOSIM revealed that the pathway profiles between the two diseases were similar (**Supplementary Figure S4B**) (*R* = 0.001, *P* = 0.395). Functional assignment of CDS clusters based on VFDB and MvirDB was performed to identify the virulence factor composition of each disease. In VFDB, ATP-dependent Clp protease proteolytic subunits were detected most frequently (PI = 1.414 ± 0.256%, PT = 1.302 ± 0.253%, mean ± standard error) in both diseases (**Supplementary Table S9**). In MvirDB, proteins found in conjugative transposons were the most prevalent in both diseases (PI = 4.038 ± 2.378%, PT = 3.959 ± 2.560%) (**Supplementary Table S10**). The virulence factor profiles of the two diseases were similar based on dendrogram and PCoA plots, which was supported by an ANOSIM (VFDB: *R* = −0.004 and *P* = 0.512, MvirDB: *R* = −0.0001 and *P* = 0.384) (**Supplementary Figures S5A–D**). The Wilcoxon tests showed no significant difference in the read abundance of virulence factors annotated against VFDB and MvirDB between the two diseases. The virulence factor profiles assigned using the above two databases revealed that the virulence factor composition of the two diseases was similar. The details of the read evaluation based on KEGG and virulence factor databases are shown in **Supplementary Result S5**.

### Correlation Between Clinical Indices and Read Abundance of CDS-Derived Taxa

To clarify the species correlated with the clinical parameters, Spearman rank correlation coefficient was calculated to determine the correlation between read abundance of the CDS-derived taxa and clinical indices including PD, RBL, and presence of KM. There were no significant correlations between the species and the three clinical indices in peri-implantitis, while a significant negative correlation between the abundance of *Corynebacterium matruchotii* and RBL was observed in periodontitis (*p* < 0.001, q = 0.028).

### Comparison of Taxonomic Profiles, Microbial Activity, and Co-Occurrence Networks Between Metagenome and Metatranscriptome

**Figure 1:**
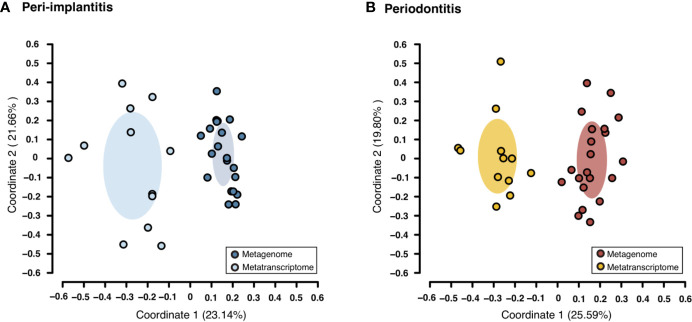
Taxonomic profile derived from genes comparing the metagenome and metatranscriptome. Principal coordinate analysis (PCoA) was carried out for the dissimilarity matrix value of 1—Spearman’s coefficient. The CDS and mRNA abundances from only species detected in both 16S and mRNA region analyses in the same subject were used in this analysis. **(A)** PCoA plots among peri-implantitis samples compared between DNA (blue circles) and RNA (light blue circles), **(B)** among periodontitis samples compared between DNA (red circles) and RNA (light red circles).

**Figure 2:**
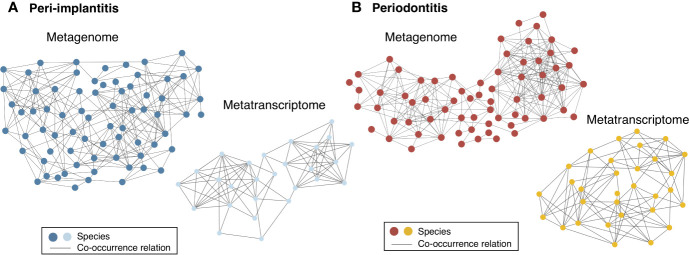
Comparison of co-occurrence networks of taxonomic profiles between metagenome and metatranscriptome. Species which were present in more than 50% of individuals in each group were used for co-occurrence network analysis. All networks are shown with each species and co-occurrence relationship indicated by a node and an edge, respectively. **(A)** Co-occurrence network profile-based taxonomic profile derived from functional genes among peri-implantitis samples comparing metagenome (blue circles) and metatranscriptome (light blue circles) results and **(B)** among periodontitis samples comparing metagenome (red circles) and metatranscriptome results (light red circles).

Both metagenomic and metatranscriptomic analyses were used to compare taxonomic, pathway, and virulence factor profiles in microbiota between peri-implantitis and periodontitis samples. At first, we compared the taxonomic profiles derived from CDS read abundance in the metagenome analysis and from mRNA read abundance in the metatranscriptome analysis. PCoA and ANOSIM showed that the composition of species taxa derived from metagenome and metatranscriptome was not similar within each peri-implantitis and periodontitis group (**Figures 1A, B**; R = 0.505, *P* = 0.001 in peri-implantitis and R = 0.348, *P* = 0.001 in periodontitis). Co-occurrence coefficient values were calculated using SparCC program (**Supplementary Tables S11**). A co-occurrence network analysis using the same method for both DNA and RNA showed 72 and 27 nodes for peri-implantitis DNA and RNA, respectively. In contrast, 68 and 31 nodes for periodontitis DNA and RNA samples, respectively, were found. In both diseases, most nodes included in the metatranscriptomic network also existed in the metagenomic network (22/27 nodes in peri-implantitis, 26/31 nodes in periodontitis). Peri-implantitis network density was 0.094 and 0.248 for DNA and RNA, respectively. In periodontitis, the network density was 0.115 and 0.228 for DNA and RNA, respectively. In both peri-implantitis and periodontitis, the number of nodes of RNA decreased compared to those of DNA, while the values of the network density of RNA increased compared to those of DNA. These results showed that the RNA co-occurrence network was more condensed than that of DNA (**Figures 2A, B**).

**Figure 3:**
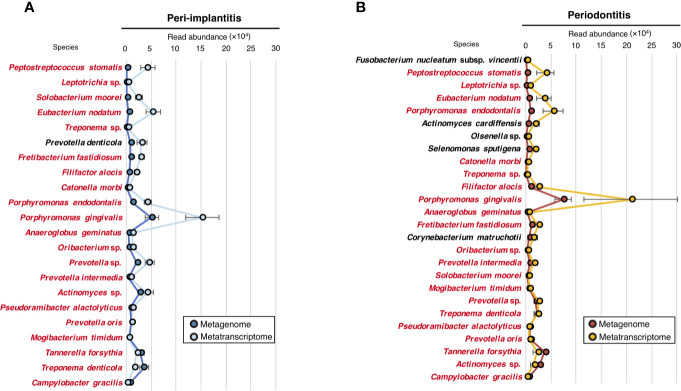
Microbial activity. **(A)** RNA/DNA ratio of species taxa identified in peri-implantitis. **(B)** RNA/DNA ratio of species taxa identified in periodontitis. Species taxa common to both peri-implantitis and periodontitis are indicated by red text.

To evaluate the microbial activity of each taxon, we only selected taxa that were commonly detected in both metagenomic and metatranscriptomic analyses and calculated the RNA/DNA ratio (**Supplementary Table S12**). The numbers of taxa that were common to DNA and RNA were 22 and 26 for peri-implantitis and periodontitis, respectively. Among them, 21 taxa were found in both diseases. The RNA/DNA ratio of *Peptostreptococcus stomatis* was highest in peri-implantitis, followed by *Leptotrichia* sp. and *Solobacterium moorei*. Furthermore, *Prevotella denticola* was only detected in peri-implantitis sites. On the other hand, *Fusobacterium nucleatum* subsp. *vincentii* had the highest ratio in periodontitis, followed by *P. stomatis* and *Leptotrichia* sp. (**Figures 3A, B**).

**Figure 4:**
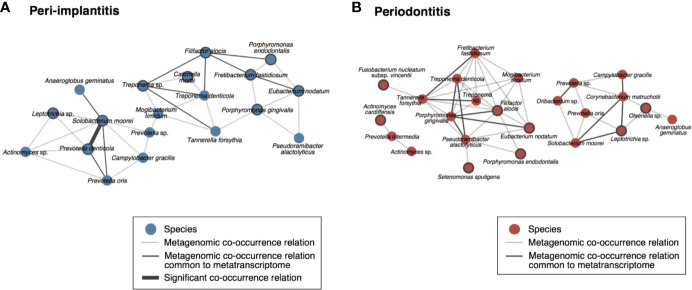
Co-occurrence networks based on species taxa common to metagenomic and metatranscriptomic analyses in **(A)** peri-implantitis and **(B)** periodontitis. All metagenomic interactions are shown by a gray line and metatranscriptomic interactions common to the metagenome are indicated by a black line. Active taxa (RNA/DNA ratio >3) are indicated by bold circles, and interactions with significant co-occurrence are indicated by bold black lines.

To visualize the co-occurrence network involving the microbial activity of each taxon, we calculated the SparCC values using only the read abundance of taxa which were found in both analyses for each metagenomic and metatranscriptomic analysis (**Supplementary Tables S13**). In peri-implantitis, eleven co-occurrence correlations were common to DNA and RNA analyses, and one of them was also significantly correlated in both analyses. Four active taxa were specific to peri-implantitis (*P. denticola*, *S. moorei*, *Porphyromonas gingivalis*, and *Fretibacterium fastidiosum*) and two taxa, *P. denticola* and *S. moorei*, were significantly correlated with each other in both metagenomic and metatranscriptomic networks and connected to four or more taxa in the co-occurrence network (**Figure 4A**). In periodontitis, although nine co-occurrence correlations were common to the DNA and RNA analyses, none showed significant correlation. Four active taxa (*F. nucleatum* subsp*. vincentii*, *Olsenella* sp., *Selenomonas sputigena*, and *Actinomyces cardiffensis*) were specific to periodontitis samples (**Figure 4B**).

### Difference of Gene Activity in Pathway and Virulence Factor Profiles Between Peri-Implantitis and Periodontitis

**Figure 5:**
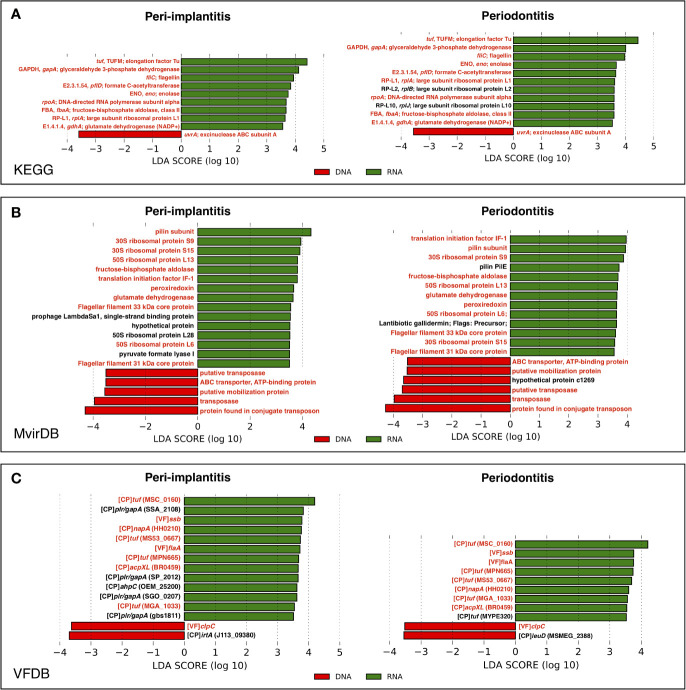
LEfSe analysis comparing peri-implantitis and periodontitis samples based on mRNA indicating enriched genes associated either with the metatranscriptome (green) or metagenome (red). Genes with significant differences common to both diseases are indicated in red. LEfSe analysis based on **(A)** KEGG, **(B)** MvirDB, and **(C)** VFDB mRNA profile assignment.

In order to determine which genes were enriched in either the metagenome or metatranscriptome, the comparison of pathway and virulence factor profiles based on KEGG, MvirDB, and VFDB using LEfSe analysis were performed. These results showed that in peri-implantitis, nine, 15, and 13 genes were significantly enriched in RNA, respectively, whereas one, five, and two genes were significantly enriched in DNA, respectively. Four out of nine RNA-enriched genes specific to peri-implantitis were plasmin receptor/glyceraldehyde-3-phosphate dehydrogenase (GAPDH) (*plr/gapA*) genes. In periodontitis, 11, 13, and nine genes were significantly enriched in RNA, respectively, whereas one, six, and two genes were significantly enriched in DNA, respectively. Transposon-related genes were enriched in DNA in both peri-implantitis and periodontitis. All genes statistically enriched in DNA or RNA are shown in **Figures 5A–C**.

## Discussion

The present study investigated microbial and gene activity in peri-implantitis and periodontitis by comparing metagenomic and metatranscriptomic analyses and succeeded in narrowing down the candidate causative bacteria and genes. *S. moorei* and *P. denticola* were highly active and were core species taxa specific to peri-implantitis in the co-occurrence network. Additionally, the activity of *plr/gapA* genes was higher in peri-implantitis than periodontitis. These results provide information on novel biomarkers for risk prevention and analytical support for the treatment of peri-implantitis. One recent review also mentioned that microbiological studies based on next-generation sequencing technologies can help to reveal the microbial pathogenesis of peri-implantitis and to develop improved preventative, diagnostic, and therapeutic strategies (Belibasakis and Manoil, 2020). One limitation of this study is that different subjects were used for each type of analysis. For analyzing and comparing the data of these two methods, it would have been ideal to use the samples from the same subjects at the same time. In addition, the samples were only collected from the deepest peri-implant sulcus and periodontal pockets, and the amount of plaque was too small to prepare both DNA and RNA samples. Hence, we utilized the metatranscriptomic data from a database in the present study. Since the speed of DNA degradation is slower than that of RNA, the results of the metagenomic analysis might have been influenced by the dead bacteria and the untranscribed genes (Moeseneder et al., 2005). Therefore, if the DNA and RNA samples were obtained from the same subjects, the result of the analysis would be similar to the results obtained in this study. Another limitation of this study was that samples were taken only from the deepest pocket. This might cause the limitation for amounts of collected samples and number of observed species.

We had previously performed 16S rRNA gene sequencing and showed that three among 44 species showed a significant difference between peri-implantitis and periodontitis in 20 subjects (Maruyama et al., 2014). Moreover, two previous studies that analyzed peri-implantitis and periodontitis microbiota of the same subjects, enrolled seven and 18 subjects (Schaumann et al., 2014; Yu et al., 2019). Regarding these previous studies, we decided to enroll a similar number of subjects with peri-implantitis and periodontitis in this study and considered that the number of subjects in this study was adequate. One review article mentioned the importance of performing analysis using samples obtained from the same subjects to compare microbial features between sites with different diagnoses (Sahrmann et al., 2020). Therefore, we believe that it is worth comparing peri-implantitis and periodontitis in the same oral cavity. Wu et al. (2016) showed that there was no significant difference in microbial composition between former smokers and never smokers. Hence, we included the five former smokers in this study. In the present study, the mean functional duration of the implants was 7.57 ± 4.99 years, and the shortest functional duration was one year in only one subject. Derks et al. (2016) reported that the progression of peri-implantitis was in a non-linear, accelerating manner, and that the onset occurred early. Hultin et al. (2002) and Becker et al. (2014) also investigated the microbiome of peri-implantitis, and the implants which were functional for at least one year were enrolled in their study. We referred to these reports and set the same criteria. The mean functional duration was similar to that in other studies analyzing the microbiome of peri-implantitis (da Silva et al., 2014; Maruyama et al., 2014; Zheng et al., 2015).

Although no species showed a significantly different abundance between peri-implantitis and periodontitis in this study, the microbial composition and diversity were slightly different between periodontitis and peri-implantitis, based on the sequence determination of the 16S rDNA, similar to the results of the previous reports (Koyanagi et al., 2010; Koyanagi et al., 2013; Maruyama et al., 2014; Schaumann et al., 2014; Yu et al., 2019). The results of the present study indicate that the presence of specific species in peri-implantitis might lead to an increase in the Shannon index. Although 16S rDNA sequencing provides the taxonomic abundance and classification, it is known that the abundance of microbial species derived from functional genes based on mRNA is different from the taxonomic abundance based on the 16S rRNA region (Shiba et al., 2016; Funahashi et al., 2019). In the present study, metagenomic analysis demonstrated that no species significantly differed in abundance between peri-implantitis and periodontitis. Since it is known that the microbiological community is site-specific, we collected the samples from only the deepest pocket to accurately analyze the correlation between microbial composition and clinical parameters. The sampling method has been commonly reported among published papers (Koyanagi et al., 2010; Maruyama et al., 2014; Araújo et al., 2015; Hernández-Vigueras et al., 2016). Only one species, *C. matruchotii*, was negatively correlated with RBL in periodontitis. *C. matruchotii* contributes to dental calculus formation by producing proteolipid inducing hydroxyapatite formation (van Dijk et al., 1998). It is known that calculus formation occurs in the early stage of periodontitis, and the progression of periodontitis is induced by adherence of the periodontal pathogen on the surface of the calculus. We considered that *C. matruchotii* was involved in the initiation of calculus formation, while the amount would be decreased by the subsequent changes in the periodontal pocket environment and microbiome.

Moreover, it was demonstrated that microbial potential was different from its expression by comparison of the metagenomic and metatranscriptomic analyses (Belstrom et al., 2017). For this reason, we compared the taxonomic profiles derived from functional genes to clarify the differences between the two diseases. The results indicate that microbial potential derived from putative functional genes differed from its expression.

Furthermore, we attempted to clarify the differences between the two diseases in network structures constructed from these data. The networks constructed with metagenomic data including factors such as dead bacteria and untranscribed genes seemed to be more complex than those from metatranscriptomic data (Moeseneder et al., 2005). Interestingly, the metatranscriptomic network density was higher than that of the metagenome in both diseases. This result likely reflects the high density of network structures at the metatranscriptome level due to the close correlation between viable bacterial species during the transcription process.

We assumed that microbial species common to RNA and DNA were important species that exist from the early stage throughout the progression of the disease. Thus, we calculated the RNA/DNA ratio which indicates the microbial activity to identify the core species of the disease during the early stage and progress. Focusing on these bacteria in co-occurrence networks of peri-implantitis, the present results suggest that *P. denticola* and *S. moorei* potentially play an important role in the co-occurrence network of peri-implantitis. Previous studies also reported that low-abundance of *S. moorei* and *P. denticola* was detected in peri-implantitis sites (Tsigarida et al., 2015; Zheng et al., 2015). *S. moorei*, a gram-positive anaerobic bacillus identified by Kageyama and Benno (2000), has been associated with diseases such as colorectal cancer (Thomas et al., 2019), bacteremia (Pedersen et al., 2011), and peri-implantitis. *P. denticola* is an obligately anaerobic, non-motile, non-spore forming, rod-shaped bacterium (Shah and Collins, 1990). It was reported that *P. denticola* was a risk indicator of periodontal disease based on a microarray analysis of 16S rDNA (Lourenco et al., 2014). The present study also shows that the prevalence of *P. denticola* in DNA is high, but that of RNA is very low at periodontitis sites. We assume that this result indicates that *P. denticola* mainly existed in the early stage of periodontitis but not much in the progression stage of the disease. *P. denticola* was more read abundance based on both DNA and RNA at peri-implantitis sites than periodontitis sites. Cortés-Acha et al. (2017) examined the microbiota surrounding the implants which were exposed to the oral cavity for 14 days, and found that *Prevotella* was the most common genus. Particularly, *P. denticola* was more overrepresented in subjects with a history of periodontitis, than in subjects without a history of periodontitis. The results showing high read abundance and prevalence of *P. denticola* at peri-implantitis sites in the present study were consistent with the results of their study. *P. denticola* was a core species that existed from the early stage throughout the progression of the disease due to the high prevalence of *P. denticola* in DNA and RNA and its high RNA/DNA ratio in peri-implantitis. The keystone pathogen hypothesis (Hajishengallis et al., 2012) supports the importance of a low-abundance microbial species in remodeling a normal microbiota community into a microbial imbalance (dysbiosis). Therefore, we should also focus on low-abundance and high-activity bacteria. Carlén et al. (2001) reported that the adhesion of bacteria depends on their surface characteristics. In addition, Fürst et al. (2007) reported that early colonization patterns differed between the implant and tooth surfaces. The bacterial difference between peri-implantitis and periodontitis sites in this study might be related to the difference in surface characteristics.

Some bacteria were detected in both DNA and RNA and existed in co-occurrence networks of periodontitis. Interestingly, the present study implies the importance of the red complex and its correlation at the DNA level, but not at the RNA level. The red complex species (*P. gingivalis*, *T. forsythia*, and *T. denticola*) were proposed as important species in periodontitis by Socransky et al. (1998). However, these species did not show high activity in periodontitis and all co-occurrence relations among red complex species were only observed in the network based on metagenomic data. On the other hand, only a co-occurrence relationship among *P. gingivalis* and *T. denticola* was observed in the network based on metatranscriptomic data. This result suggests that these microbial species have the potential to correlate with each other, while the correlation *in situ* was limited. As for red complex of peri-implantitis, only two correlations between *T. forsythia* and *T. denticola*, and *P. gingivalis* and *T. forsythia* in the metagenomic co-occurrence network were observed. Additionally, all correlations between red complex were not observed in the metatranscriptomic co-occurrence network. For this reason, the red complex might also not be important in either the early stage or progression of peri-implantitis. Regarding the network structures, the network density value of peri-implantitis was higher than that of periodontitis, implying that the network structure of peri-implantitis was more closely connected than that of periodontitis. For this reason, it is considered that the network structure of peri-implantitis is more robust than that of periodontitis, and this characteristic of the microbiome of peri-implantitis brings more potential for resistance to clinical treatment than periodontitis.

Transcriptional regulation of bacterial gene expression is controlled at the transcriptional level by the environment (Guiney, 1997; Green et al., 2014). Therefore, we examined the differences in pathway and virulence factor profiles between the metagenome and metatranscriptome to investigate active genes. Analysis based on the virulence factor database revealed that *plr/gapA*-related genes were highly transcribed in peri-implantitis than in periodontitis. These genes are known to be derived from *Streptococcus* spp. (Winram and Lottenberg, 1996; Terao et al., 2006). However, the species were not identified as highly active species in this study, suggesting that some bacteria expressing *plr/gapA*-related genes might contribute to the pathogenesis of peri-implantitis. In addition, the analysis based on the pathway database revealed that the GAPDH-related gene was highly transcribed in both, peri-implantitis and periodontitis. The result suggests that the microbial pathogenicity of GAPDH might be attributed to the onset and progression of both diseases. Although GAPDH is known to be an important enzyme in glycolysis, it has been reported that this protein has various microbial virulence functions. Maeda et al. (2004) reported that the GAPDH derived from plaque-forming bacteria bound to *P. gingivalis* fimbriae contributes to the colonization of *P. gingivalis.* Furthermore, Seidler and Seidler (2013) reported that pathogenic GAPDH inhibits phagocytosis of the host immune system, and also escapes from immune surveillance. These pathogenic mechanisms may help establish a disease-specific pathology such as faster disease progression than that of periodontitis. This gave us the suggestion that bacterial GAPDH may be a potential biomarker for control of the microbiome of peri-implantitis. Indeed, another study revealed that a recombinant GAPDH protein might be a potential inhibitor of *P. gingivalis* coaggregation (Nagata et al., 2009). However, GAPDH is expressed not only by bacteria but also by host cells; thus, further *in vitro* research is needed to reveal the details of the role of GAPDH.

In summary, the structure of transcription level was distinct from that of potential level in terms of co-occurrence network. A small number of core species in the co-occurrence networks and high-expression genes were observed to be specific to peri-implantitis. It is likely worth comparing the metagenomic and metatranscriptomic data, although the subjects for the metagenomic and metatranscriptomic analysis were different in this study. Our findings provide information on a novel biomarker and disease-specific co-occurrence network for peri-implantitis. Such disease-specific microbial characteristics distinguish clinical features between peri-implantitis and periodontitis.

## Data Availability Statement

The datasets generated for this study can be found in the DNA Data Bank of Japan (DDBJ) with the following accession numbers: 16S rDNA sequencing (DRA010104) and metagenomic analysis (DRA006832).

## Ethics Statement

The studies involving human participants were reviewed and approved by Ethics Committee of Tokyo Medical and Dental University (D2015-535). The patients/participants provided their written informed consent to participate in this study.

## Author Contributions

KK and TS performed the experiments and wrote the first draft of the manuscript. TW, TN, TK, SKat, MShim, MShib, YT, and TI assisted with the experiment and reviewed the manuscript. KK, TS, TN, TK, MShim, and YT performed the sample collection. TW and TS provided expertise on microbiome analysis and designed the study. YT, SKas, and TI supervised analyses and the writing of the manuscript. Correspondence and material requests should be addressed to TS or TW. All authors contributed to the article and approved the submitted version.

## Funding

This work was supported by the Japan Society for the Promotion of Science (19K10164 to TK, 17H06662 and 19K19016 to TS, 17K11981 to YT).

## Conflict of Interest

The authors declare that the research was conducted in the absence of any commercial or financial relationships that could be construed as a potential conflict of interest.
