# Supplementary material for: Discriminating Microbial Community Structure Between Peri-Implantitis and Periodontitis With Integrated Metagenomic, Metatranscriptomic, and Network Analysis
Source: Front Cell Infect Microbiol. 2020 Dec 11;10:596490. doi: 10.3389/fcimb.2020.596490 (PMC7793907; doi:10.3389/fcimb.2020.596490)
Supplement: Supplementary file 1 [file DataSheet_1.docx]

Supplementary Material

## Supplementary Methods

# The detail of radiographic examination and measurement of radiographic bone loss (RBL)

Standardized intra-oral periapical digital (CS 7600, Carestream Health, Rochester, NY, USA) and/or analog (Insight Dental Film; Eastman Kodak Company, SP, Tokyo, Japan) radiographs were obtained by parallel technique. RBL was calculated by measuring the linear distance from a straight line connected by the mesiodistal bone ridge to the bottom of the bone defect using ImageJ software image processing (V1.51, National Institutes of Health, Bethesda, MD, USA); all calculations were made by the same examiner.

# Sample preparation and data analysis for 16S ribosomal DNA (16S rDNA) sequencing

The DNA sample of the V3-V4 region was amplified with HiFi hot fidelity primer (New England Biolabs, USA) and the following primers: forward (5'- TCGTCGGCAGCGTCAGATGTGTATAAGAGACAGCCTACGGGNGGCWGCAG -3’) and reverse (5-' GTCTCGTGGGCTCGGAGATGTGTATAAGAGACAGGACTACHVGGGTATCTAATCC -3’). The purity of amplified products was assessed with an Agilent 2100 Bioanalyzer (Agilent Technologies, Santa Clara, CA, USA) and they were purified with the ProNex Size-Selective Purification System (Promega, Madison, WI, USA). These purified products were attached to Illumina sequencing adapters using Nextera XT index primer and purified with the ProNex Size-Selective Purification System (Promega, Madison, WI, USA) again. Each purified sample was assessed for quantity with quantitative PCR (qPCR) using the KAPA Library Quantification Kits (KAPA Biosystems, Wilmington, MA, USA) and the quantity of each sample was normalized to maintain the same concentration of the pooled library. The final library was combined with the PhiX Control Library (v3) (Illumina) to improve the diversity of base calls and sequencing data was obtained for 16S rDNA by using the MiSeq V3 reagent kit (Illumina, San Diego, California, USA). Demultiplexed forward and reverse reads obtained by MiSeq were processed according to the recommended parameters of the Illinois Mayo Taxon Organization from RNA Dataset Operations (IM-TORNADO) (Jeraldo et al., 2014) pipeline for 300 bp reads. The demultiplexed sequence reads were quality filtered using Trimmomatic v.0.30 software (Bolger et al., 2014) in the pipeline with following parameters: ILLUMINACLIP:2:20:10, LEADING:15, TRAILING:15 SLIDINGWINDOW: 4:15. Shorter than 75% of the original read length reads were removed with recommended parameter for 300 bp: MINLEN 225. Filtered read pairs are grouped into R1 read and R2 read. R1 read was keep whole read length, R2 read was trimmed down to 240 bp (R1_TRIM=300, R2_TRIM=240). Only paired reads were dereplicated, building clusters of reads with 100% similarity and annotated with cluster size. Shorter than the cutoff length reads and singleton reads were removed to guarantee the use of high quality reads when finding the OTU representatives. OTU representatives were employed a k-mer-based approach for taxonomy assignment using the Ribosomal Database Project naive Bayesian classifier with a threshold of 80% bootstrap confidence. Each OTUs was assigned at the species level with the Human Oral Microbial database (HOMD) (Chen et al., 2010) at 97% sequence identity.

# Sample preparation and data analysis for metagenomic sequencing

Metagenomic library preparation was performed using a Nextera XT DNA library preparation kit (Illumina Inc., CA, United States) according to the manufacturer’s protocol. Briefly, after tagmentation of normalized samples, samples were purified using AMpure XP beads (Beckman Coulter, Brea, CA, USA) and their quality was checked with an Agilent 2100 Bioanalyzer using a High Sensitivity DNA kit (Agilent Technologies, Santa Clara, CA, USA). Subsequently, the quantity of each purified sample was assessed by qPCR using KAPA Library Quantification Kits and the quantity of each sample was normalized to maintain the same concentration as that of the pooled library. The sequencing libraries were combined with the PhiX Control Library (v3) (Illumina) to improve base call diversity and metagenome data were obtained using a MiSeq V2 reagent kit (Illumina, San Diego, CA, USA) running 2 × 250 bp paired-end reads. Metagenomic sequencing data preprocessing was conducted with the same each analysis tool and parameters that were previously reported (Shiba et al., 2016). The obtained metagenomic sequencing data were filtered by Trimmomatic v.0.32 software (Bolger et al., 2014) and Eukaryote origin reads were removed using DeconSeq v.0.4.3 software (Schmieder and Edwards, 2011). The remaining paired and unpaired reads were assigned based on the Metagenomics Rapid Annotation using the Subsystem Technology (MG-RAST) pipeline (Meyer et al., 2008) to reveal the pathway profile with the Kyoto Encyclopedia of Genes and Genomes (KEGG) database (Kanehisa and Goto, 2000). Subsequently, paired reads were combined with each read pair using fastq-join (Aronesty, 2013), and clusters were formed by using the Cluster Database at High Identity with Tolerance (CD-HIT) program. The putative rRNA genes originating were presumed using ARB-SILVA (release 119). Those sequences with nucleotide similarities to those were extracted as putative 16S rRNA gene reads and removed (Shi et al., 2009). The remaining cluster (putative CDS cluster) was assigned based on the Virulence Factors Database (VFDB; as of December 23, 2016) and the Microbial Virulence Database (MvirDB; as of December 20, 2016) to reveal the microbial virulence factors. The representative sequences of the mRNA clusters were subjected to BlastX searching against these 2 databases. We also examined the genetic profile and mRNA-derived bacterial species using the CDS clusters assigned based on the National Center for Biotechnology Information Non-Redundant Protein Database (NCBI nr; as of January 10, 2017). Metagenomic sequencing data were deposited in the DNA Data Bank of Japan (DDBJ; http://www.ddbj.nig.ac.jp/) under accession number DRA006832.

# Details of metatranscriptomic analysis

Metatranscriptomic data were obtained from the DDBJ (DRA003492). All sequencing data preprocessing was conducted following a previous method (Shiba et al., 2016). The number of reads used in the analysis, the taxonomic profile based on NCBI nr database, the functional profile based on KEGG, MvirDB, VFDB, and the SparCC values were consistent with those of previous reports. All the details of data preprocessing methods and results were shown in previous study (Shiba et al., 2016).

# Supplementary Results

# Summary of sequence reads

A total of 16,230,298 and 53,435,642 sequence reads were obtained from 16S rDNA sequencing and metagenomic sequencing, respectively. The average numbers of obtained reads per sample for 16S rDNA sequencing and metagenomic sequencing were 386,436 ± 256,830 and 1,272,277 ± 227,632 (mean ± SD), respectively. The number of sequence reads for 16S rDNA sequencing and metagenomic sequencing did not differ significantly between the peri-implantitis and periodontitis samples. A total of 1,134,416 and 36,793,385 preprocessed reads were obtained from 16S rDNA sequencing and metagenomic sequencing, respectively. The average number of obtained reads per sample for 16S rDNA sequencing and metagenomic sequencing was 27,010 ± 19,703 and 876,033 ± 214,233 (mean ± SD), respectively. The number of preprocessed reads of both 16S rDNA sequencing and metagenomic sequencing did not also differ significantly between peri-implantitis and periodontitis samples (Supplementary Table S2).

# Taxonomic profile and diversity based on 16S rDNA sequencing

At the species level, *Fusobacterium nucleatum* subsp. *vincentii* was the most dominant bacterial species for both peri-implantitis (14.67% ± 10.75%) and periodontitis (17.89% ± 10.24%). In peri-implantitis samples, *Porphyromonas gingivalis* (10.34% ± 14.32%) and *Porphyromonas endodontalis* (3.20% ± 4.94%) were the next most dominant species. In periodontitis samples, *P. gingivalis* (16.61% ± 16.98%) and *Streptococcus* sp. (2.68% ± 4.19%) were the next most dominant species (Supplementary Figure S2 and Supplementary Table S4).

# Taxonomic profile based on NCBI nr database

The total number of taxa in peri-implantitis and periodontitis were 4,702 and 4,751, respectively; a total of 4,455 taxa were common to both diseases (Supplementary Table S5). Most disease-specific taxa appeared infrequently and were only detected in a few subjects. In both diseases, the most predominant taxon was *P. gingivalis* which is a known periodontal pathogen (PI = 5.60 ± 6,60%, PT = 8.46 ± 8.73%). The second most predominant taxon was *Treponema denticola*, followed by *Actinomyces* sp. in peri-implantitis. On the other hand, the second most predominant taxon was *Tannerella forsythia*, followed by *Fusobacterium nucleatum* in periodontitis (Supplementary Figure S3B).

# Putative coding domain sequence (CDS) profile based on NCBI nr database

The total numbers of CDS genes in peri-implantitis and periodontitis were 132,360 and 142,985, respectively (Supplementary Table S6 and S7). In both diseases, the most predominant functional gene was membrane protein (PI = 1.98 ± 0.51%, PT = 2.06 ± 0.54%). The second most predominant functional gene was hypothetical protein ET33_03555 (0.82 ± 0.97%), followed by Transposase (0.77 ± 0.20%) in peri-implantitis. On the other hands, the second predominant functional gene was transposase (0.91 ± 0.22 %), followed by unnamed protein product (0.82 ± 0.97%) in periodontitis.

# Pathway and virulence factor profiles based on metagenomic analysis

Metagenomics Rapid Annotation using the Subsystem Technology (Meyer et al., 2008) pipeline analysis revealed the characteristics of putative CDS reads based on KEGG (Kanehisa and Goto, 2000) pathways and showed that at KEGG Level 4 assignment, 2,091 genes were detected in both groups; of these, 1,754 genes (83.88%) were common to both diseases (Supplementary Table S8). In the VFDB, the total number of virulence factor-assigned CDS clusters in peri-implantitis and periodontitis were 5,961 and 6,363, respectively; a total of 5,185 virulence factors were common to both diseases (Supplementary Table S9). In the MvirDB, the total numbers of virulence factor-assigned CDS clusters in peri-implantitis and periodontitis were 3,355 and 3,459, respectively; a total of 2,995 virulence factors were common to both diseases (Supplementary Table S10).

**Supplementary Figures**

**
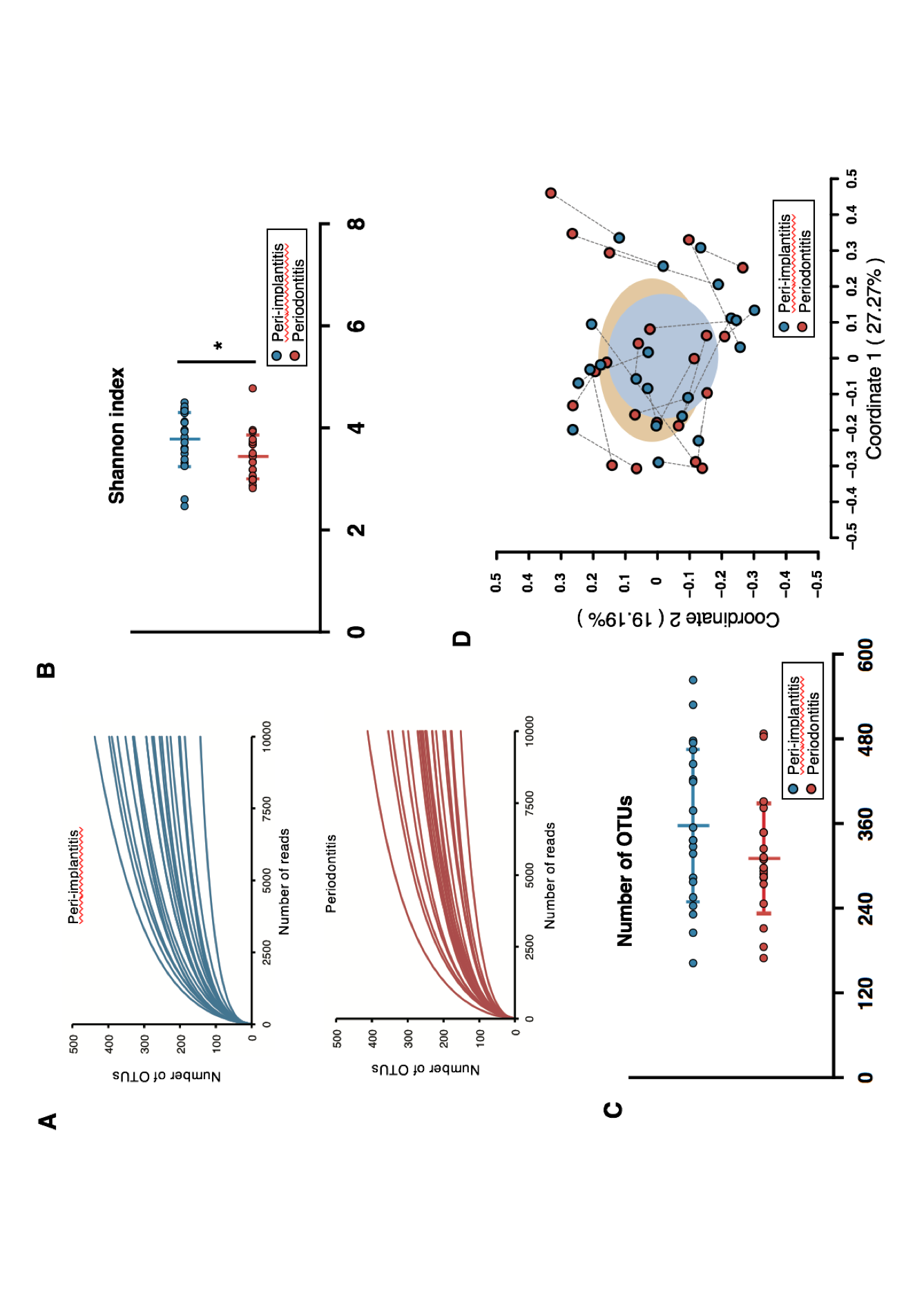
Supplementary Figure S1.** Evaluation of peri-implantitis and periodontitis microbiome composition based on 16S rDNA sequencing (n = 21). (A) Rarefaction curves, (B) Shannon index, and (C) number of observed OTUs between peri-implantitis and periodontitis areas. Error bars represent standard deviation of the mean. (D) Principal coordinate analysis (PCoA) was carried out for the dissimilarity matrix value of 1 − Spearman’s coefficient, and 21 peri-implantitis (blue circles) and periodontitis (red circles) samples were plotted with two coordinates. The mean and standard deviation in each axis are indicated by an ellipse for each disease group. Dots corresponding to peri-implantitis and periodontitis samples from the same patient are connected by a dotted line. **P* < 0.05.


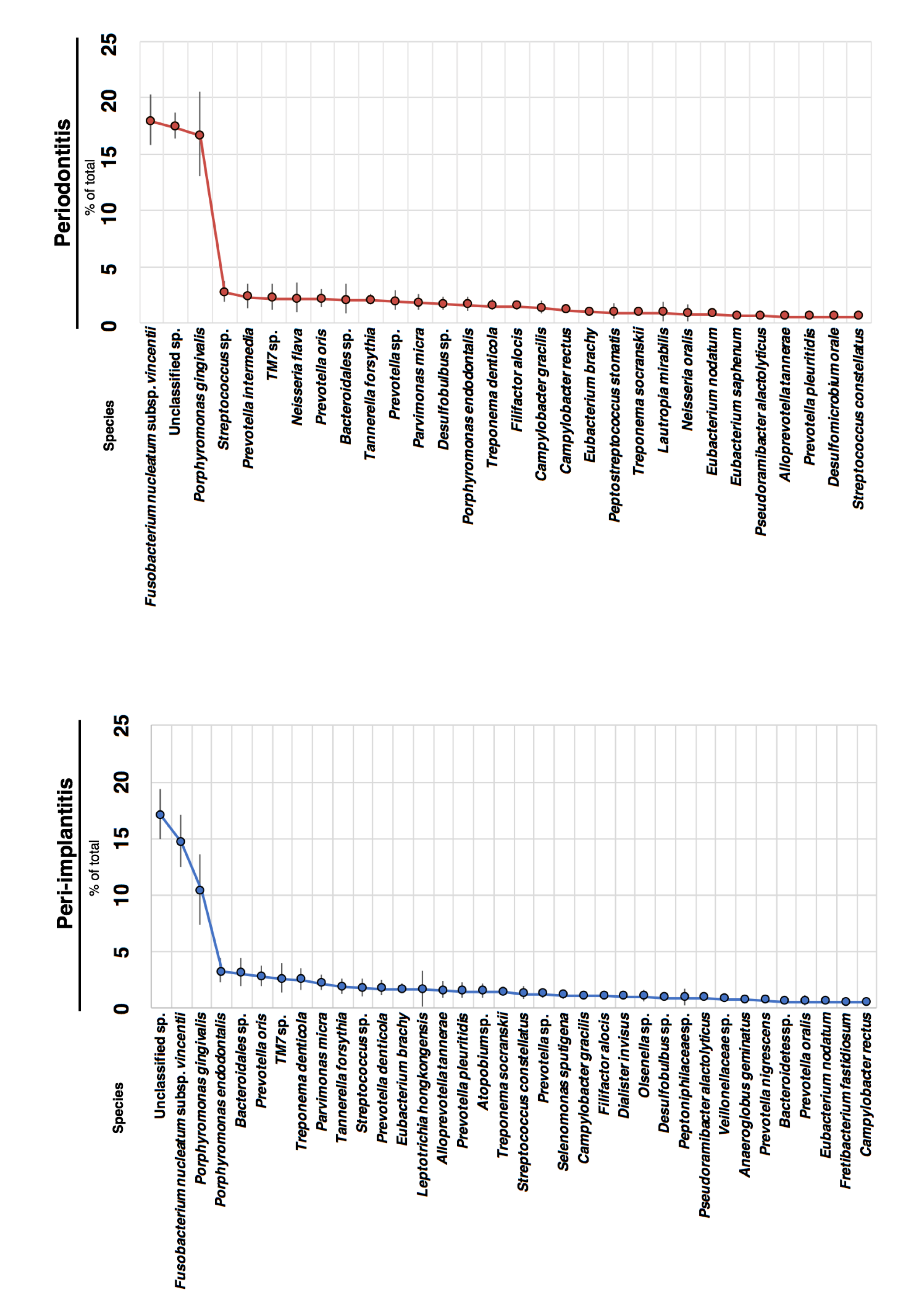
**Supplementary Figure S2.** Taxonomic profile composition based on 16S rDNA sequencing. Rank distributions of taxonomic origins for 16S rDNA clusters in peri-implantitis and periodontitis samples are shown. Mean 16S rDNA abundances of 21 samples are shown in descending order with standard error bars. Only species with ≥ 0.5% relative abundances in both diseases are shown. Error bars represent standard errors of the mean.


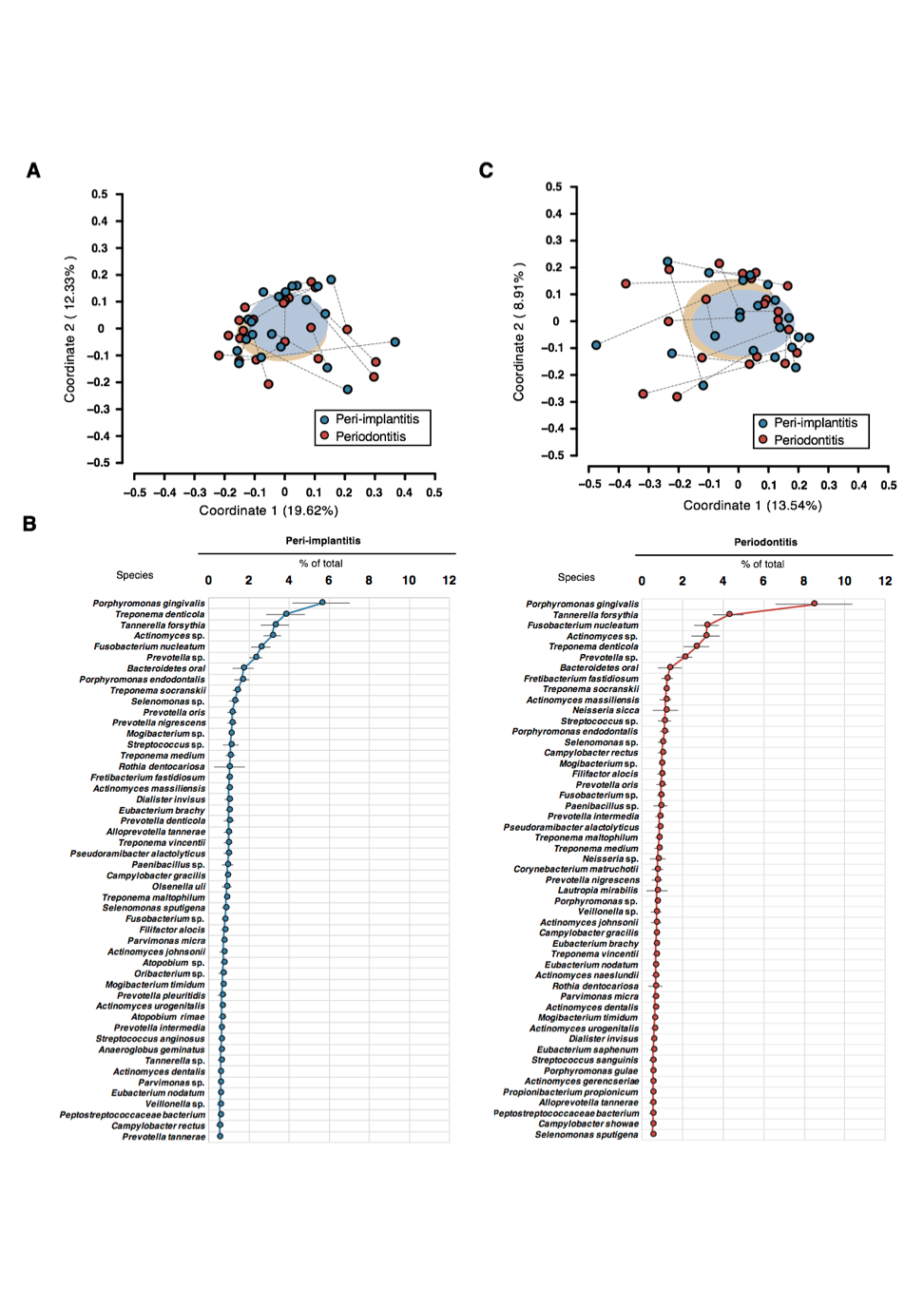


**Supplementary Figure S3.** Taxonomic and CDS profiles composition based on NCBI nr database by using metagenomic analysis. (A) PCoA plots prepared from taxonomic profiles assigned with the NCBI nr database, as described in Supplementary Figure S1D. (B) Rank distributions of taxonomic origins for CDS clusters in peri-implantitis and periodontitis samples are shown. Mean mRNA abundances of 21 samples are shown in descending order with standard error bars. Only species with ≥ 0.5% relative abundances in both diseases are shown. (C) PCoA plots prepared from CDS profiles assigned with the NCBI nr database as described in Supplementary Figure S1D.

**
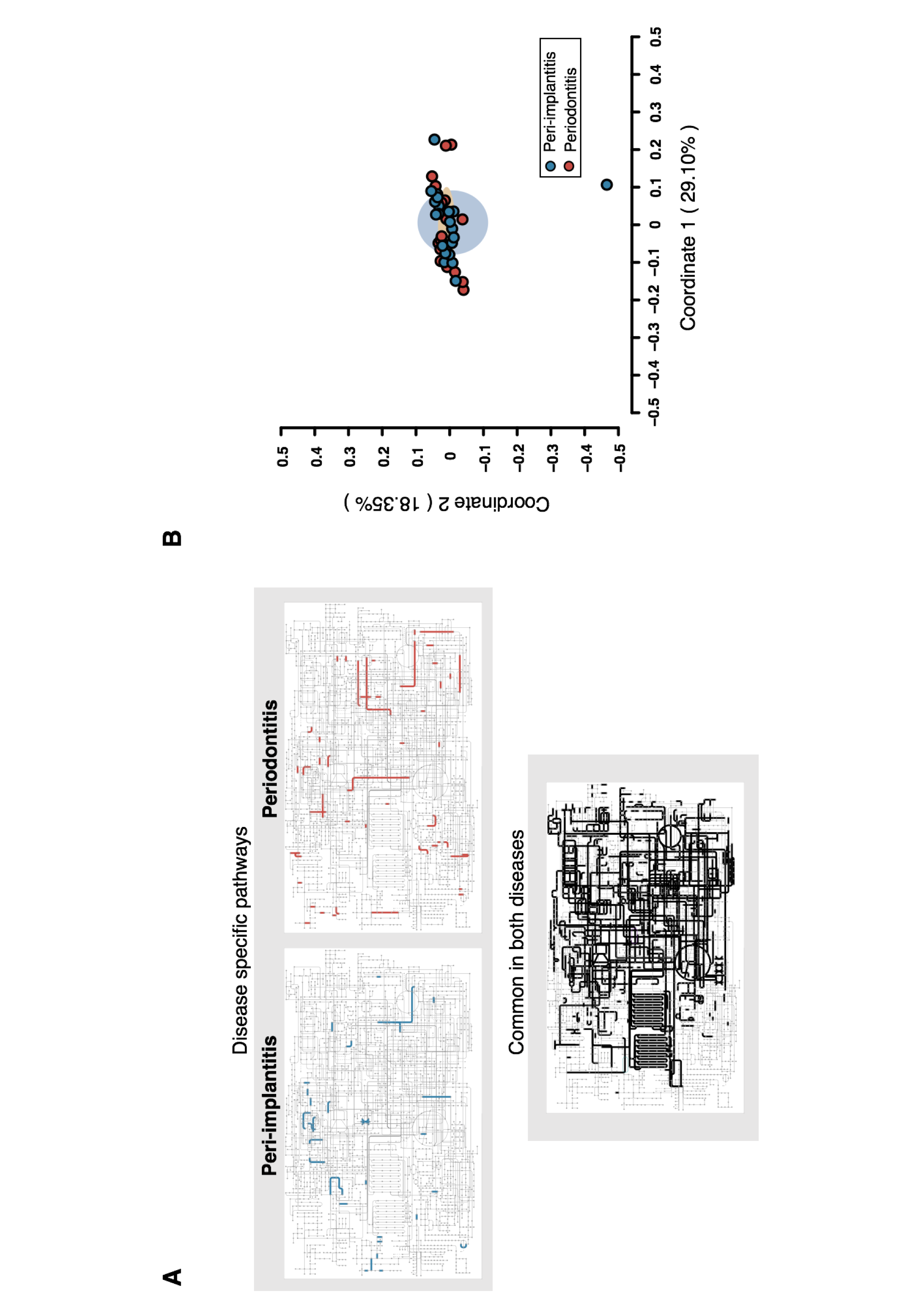
**

**Supplementary Figure S4.** KEGG pathway profiles of CDS reads clusters based on metagenomic analysis. (A) Disease-specific KEGG pathways present in any of the 21 samples for each disease and common KEGG pathways. (B) PCoA plots prepared from CDS profiles assigned using KEGG as described in Supplementary Figure S1D.

**
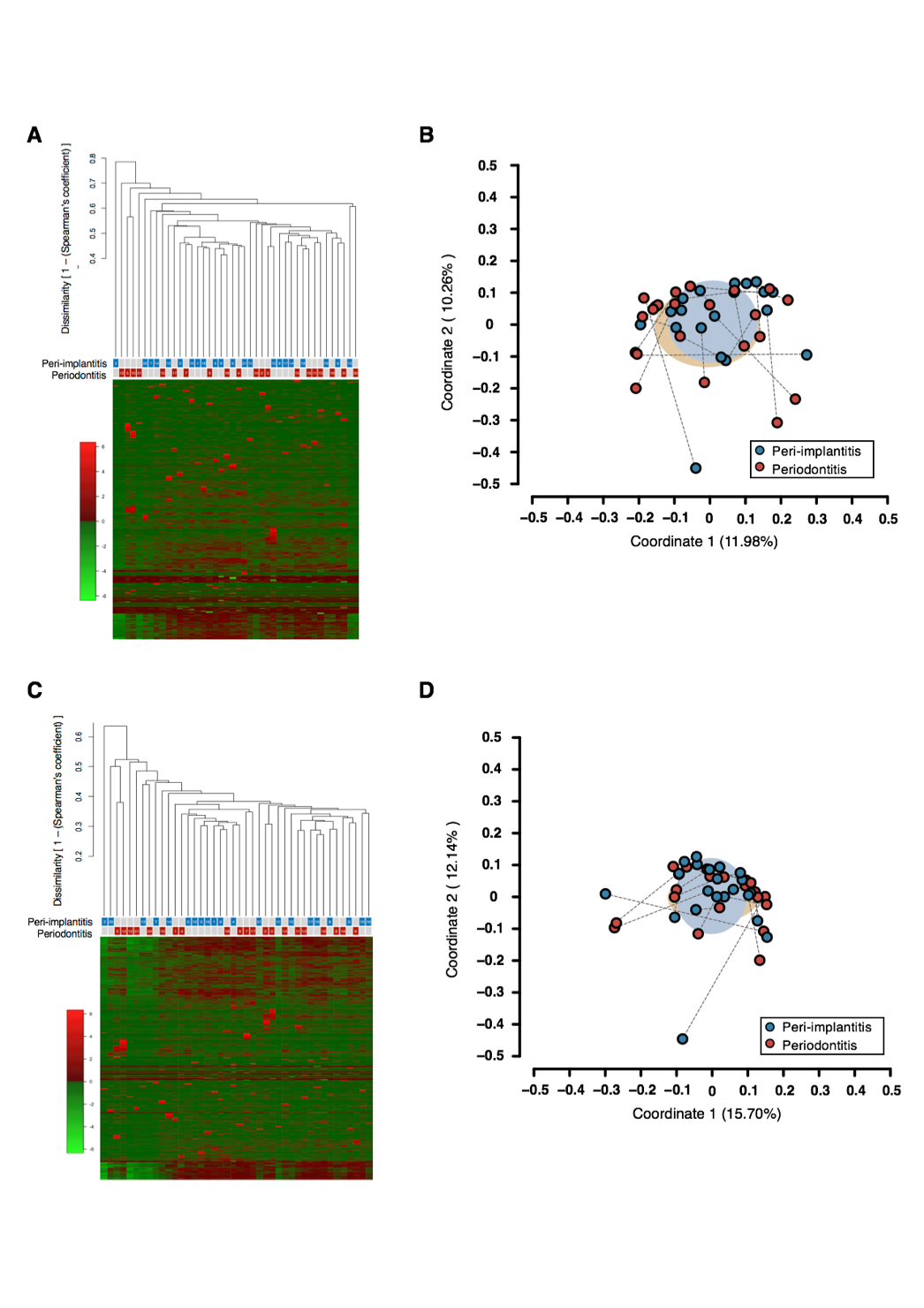
**

**Supplementary Figure S5.** Virulence factor gene profile of mRNA reads assigned to VFDB and MvirDB based on metagenomic analysis. (A) Dissimilarity values (1 − Spearman’s coefficient) were clustered using the average linkage method as shown in the dendrogram based on VFDB. Disease types and patient numbers are shown under the tree. The heat map shows the log2 CDS read abundance for each virulence factor gene as indicated by the color gradient. (B) PCoA plots based on VFDB prepared as described in Supplementary Figure S1D. (C) Dendrogram based on MvirDB. (D) PCoA plots based on MvirDB prepared as described in Supplementary Figure S1D.

**REFERENCES**

Aronesty, E. (2013). "Comparison of Sequencing Utility Programs". *The Open Bioinformatics Journal* 7**,** 1-8. doi: 10.2174/1875036201307010001.

Bolger, A.M., Lohse, M., and Usadel, B. (2014). Trimmomatic: a flexible trimmer for Illumina sequence data. *Bioinformatics* 30(15)**,** 2114-2120. doi: 10.1093/bioinformatics/btu170.

Chen, T., Yu, W.H., Izard, J., Baranova, O.V., Lakshmanan, A., and Dewhirst, F.E. (2010). The Human Oral Microbiome Database: a web accessible resource for investigating oral microbe taxonomic and genomic information. *Database (Oxford)* 2010**,** baq013. doi: 10.1093/database/baq013.

Jeraldo, P., Kalari, K., Chen, X., Bhavsar, J., Mangalam, A., White, B., et al. (2014). IM-TORNADO: a tool for comparison of 16S reads from paired-end libraries. *PLoS One* 9(12)**,** e114804. doi: 10.1371/journal.pone.0114804.

Kanehisa, M., and Goto, S. (2000). KEGG: kyoto encyclopedia of genes and genomes. *Nucleic Acids Res* 28(1)**,** 27-30. doi: 10.1093/nar/28.1.27.

Meyer, F., Paarmann, D., D'Souza, M., Olson, R., Glass, E.M., Kubal, M., et al. (2008). The metagenomics RAST server - a public resource for the automatic phylogenetic and functional analysis of metagenomes. *BMC Bioinformatics* 9**,** 386. doi: 10.1186/1471-2105-9-386.

Schmieder, R., and Edwards, R. (2011). Fast identification and removal of sequence contamination from genomic and metagenomic datasets. *PLoS One* 6(3)**,** e17288. doi: 10.1371/journal.pone.0017288.

Shiba, T., Watanabe, T., Kachi, H., Koyanagi, T., Maruyama, N., Murase, K., et al. (2016). Distinct interacting core taxa in co-occurrence networks enable discrimination of polymicrobial oral diseases with similar symptoms. *Sci Rep* 6**,** 30997. doi: 10.1038/srep30997.
